# Reconsidering Dogmas about the Growth of Bacterial Populations

**DOI:** 10.3390/cells12101430

**Published:** 2023-05-19

**Authors:** Bettina Ughy, Sarolta Nagyapati, Dezi B. Lajko, Tamas Letoha, Adam Prohaszka, Dima Deeb, Andras Der, Aladar Pettko-Szandtner, Laszlo Szilak

**Affiliations:** 1 Institute of Plant Biology, Biological Research Centre, Eötvös Loránd Research Network, H-6726 Szeged, Hungary; nagyapati.sarolta@brc.hu (S.N.); lajkodezi@gmail.com (D.B.L.); adam.proha@gmail.com (A.P.); deeb.dima@brc.hu (D.D.); 2 Doctoral School in Biology, Faculty of Science and Informatics, University of Szeged, H-6726 Szeged, Hungary; 3 PharmacoIdea Ltd., H-6726 Szeged, Hungary; tamas.letoha@pharmacoidea.eu; 4 Institute of Biophysics, Biological Research Centre, Eötvös Loránd Research Network, H-6726 Szeged, Hungary; 5 Laboratory of Proteomic Research, Biological Research Centre, Eötvös Loránd Research Network, H-6726 Szeged, Hungary; pettko-szandtner.aladar@brc.hu; 6 Szilak Laboratories Bioinformatics and Molecule-Design Ltd., H-6724 Szeged, Hungary

**Keywords:** population growth, stationary phase, exponential phase, differentiation, transition, generation time, proteomics

## Abstract

The growth of bacterial populations has been described as a dynamic process of continuous reproduction and cell death. However, this is far from the reality. In a well fed, growing bacterial population, the stationary phase inevitably occurs, and it is not due to accumulated toxins or cell death. A population spends the most time in the stationary phase, where the phenotype of the cells alters from the proliferating ones, and only the colony forming unit (CFU) decreases after a while, not the total cell concentration. A bacterial population can be considered as a virtual tissue as a result of a specific differentiation process, in which the exponential-phase cells develop to stationary-phase cells and eventually reach the unculturable form. The richness of the nutrient had no effect on growth rate or on stationary cell density. The generation time seems not to be a constant value, but it depended on the concentration of the starter cultures. Inoculations with serial dilutions of stationary populations reveal a so-called minimal stationary cell concentration (MSCC) point, up to which the cell concentrations remain constant upon dilutions; that seems to be universal among unicellular organisms.

## 1. Introduction

In the natural environment, constant bacterial growth is seldom found, in contrast to laboratory conditions, when bacteria are cultured in rich media at an optimal temperature. In a nutrition-rich medium, bacteria can sustain a continuous, relatively fast, balanced growth for a given time [1,2]. According to the literature, the characteristic bacterial growth curve pattern comprises four phases: lag, exponential growth, stationary phase, and death phase. During the lag phase, bacteria in nutrient-rich medium need to adapt to the new environment in order to start cellular metabolism; during this, there is a little or no cell reproduction that takes place. The lag duration has often been considered erratic and unpredictable [3]. At the end of the lag phase, the cell concentration starts increasing, and the population enters the exponential growth phase [4]. Since bacterial cells divide by binary fission, the growth rate of cultures is logarithmic and characterized by the number of doublings, that is, the generation time [5]. During the exponential (or logarithmic) phase, bacterial reproduction occurs at a maximal rate characteristic for the given growth condition. It is believed that the growth rate depends directly on the culture medium that is slower in nutrient-poor and faster in nutrient-rich conditions [2]. If cells in the exponential growth phase are inoculated into fresh medium, the lag phase is bypassed, and the logarithmic phase continues. Unicellular populations grown in batch culture, after a while, reach a point when the growth rate decreases, and the increase in cell number ceases; this is defined as the stationary phase. The stationary phase is characterized, usually, by a dynamic equilibrium between the numbers of dividing and dying cells, resulting in a constant cell number represented by a plateau in the growth curve [6]. The state-of-the-art is consistent, in that, when nutrients become limited or other environmental conditions restrict growth, the bacterial cultures enter the stationary phase [1,2]. However, this description does not distinguish between the metabolically active or inactive cells because it just simply refers to a culture that shows no increment in the cell number [6]. When the environment cannot maintain the population, cultures in the stationary phase accumulate toxic products of catabolism that lead to a decrease in the number of viable cells defined as the death phase. Nowadays, a fifth phase is distinguished, termed as the long-term stationary phase, in which the minority of the population could hold their viability for several weeks and even months [7]. For example, *Escherichia coli* (*E. coli*) could be maintained in batch culture for long periods of time (more than five years without the addition of nutrients) [7].

According to the state-of-the-art, the transition from the logarithmic phase to the stationary phase is driven by the σ^38^ (σ^S^)-factor, encoded by the *rpoS* gene in *E. coli* cells [8], whose transcription is repressed by the phosphorylated form of the ArcA regulator in the exponential phase [9]. The transition to stationary phase is not limited only to the activation of σ^38^, but other alterations of metabolism occur, e.g., hibernation of ribosomes regulated by RelA via the synthesis of the molecule ppGpp (alarmone) as a response to growing starvation and adaptation to long-term survival [10]. The increasing nutrient deprivation in the stationary phase induces decrement or loss of the colony forming units (CFU) in standard plating assays that can be considered as a transition to the death phase [7]. The loss of the proliferating capability raises the question of the bacterial life span and aging. Ref. [11] reported that bacteria can fail to divide on laboratory media, but they still appear viable. This phenomenon is termed viable, but not culturable (VBNC) [11,12,13,14]. The non-proliferative cells seem to be in a growth-arrested, G_0_-like state that is incompatible with colony formation on nutrient agar plates [1].

The theory that cell growth can be controlled by direct cell–cell contact mediated interaction was raised in unicellular algae [15], and it was described in *E. coli* by the CdiA-CdiB system, defined as contact-dependent growth inhibition (CDI). The growth inhibition was dependent on the growth state of the inhibitory cells, occurring in the logarithmic phase, but not in the stationary phase. Therefore, this system cannot be implicated in the development of stationary cell concentration [16,17]. CDI systems are suggested mostly to play roles in bacterial competition to hinder the growth of neighboring microbes [18].

The experiments were carried out on cyanobacteria, a well characterized strain of *Synechococcus elongatus* PCC7942 (*S. elongatus*), which is an autotrophic, photosynthetic, Gram-negative prokaryotic microorganism carrying three to eight chromosomal copies per cell, in contrast to more traditionally studied bacteria, such as *E. coli* or *Bacillus subtilis*, which typically contain only one or two complete chromosomal copies [19]. Cyanobacteria are oxygenic, photosynthetic Gram-negative bacteria, converting solar energy into chemical energy. They are the most abundant microorganisms in aquatic environments and play a key role in the global carbon cycle. It is estimated that these photosynthetic microbes are responsible for at least 50% of carbon fixation in the oceans [20]. They are usually considered as ancestors of chloroplasts in higher plants.

We acknowledge that the stationary phase could be achieved by starvation, toxins, or abiotic stress. However, we investigated the development of so-called native stationary phases in optimal conditions, which are not due to the listed ones. In normal laboratorial conditions, the depletion of nutrients occurs rarely, and the spent medium could provide enough nutrients to enable the growth of cultures and reach the normal stationary plateau more times. Here, we challenge a generally accepted view of bacterial growth in batch culture in the case of *S. elongatus*. We examined the exponential and stationary growth phases in good conditions and concluded that the stationary phase could not be due to nutrient depletion, toxins, or contact inhibition. With serial dilutions of stationary populations used for inoculations, we could define a minimal stationary cell concentration (MSCC) point. Similar results were gained from studies of other unicellular organisms, as well. Proteomic analysis sheds light on the fact that the stationary-phase cells changed their phenotype. Overall, we should reconsider the old principles of bacterial growth.

## 2. Materials and Methods

### 2.1. Cell Culture and Growth Conditions

*S. elongatus* PCC7942 cells were grown photoautotrophically in BG-11 medium [21], supplemented with 10 mM HEPES–NaOH (pH 8). The media used for the growth of *E. coli* (*Escherichia coli* K12, DH5alphaF’ (*F’/endA1 hsdR17 (r_k_^−^m_k_^+^) supE44 thi-1 recA1 gyrA (Nal^r^) relA1 D(laclZYA-argF)U169 deoR (*ϕ*80dlacD(lacZ) M15*)) were the following: LB (pepton 10 g L^−1^, yeast extract 5 g L^−1^, NaCl 10 g L^−1^), 2YT (tryptone 16 g L^−1^, yeast extract 10 g L^−1^, NaCl 5 g L^−1^), and SOC (tryptone 20 g L^−1^, yeast extract 5 g L^−1^, NaCl 0.5 g L^−1^, glucose 20 mM). The *E. coli* was cultivated in LB, unless otherwise stated. The bacteria were grown in Erlenmeyer flasks on a rotary shaker by shaking *E. coli* at 200 rpm, at 37 °C. Additionally, *S. elongatus* was rotated at 110 rpm at 29 °C under continuous illumination at 40 μmol photons m^−2^ s^−1^ white light intensity. The centrifugation of the cultures was carried out with 4000× *g* for 10 min in sterile conditions using an Eppendorf 5910 R centrifuge (Eppendorf, Hamburg, Germany).

### 2.2. Measurement of Optical Density

The optical density of the cyanobacterial cultures was determined daily by measuring OD at 580 nm in the case of *S. elongatus* and at 600 nm in the case of *E. coli* using a Nicolet Evolution 500 spectrophotometer (Thermo Electron, Cambridge, UK). In the case of *S. elongatus,* all of the OD measurements were executed at 730 and 750 nm, as well, but the curves were very similar to each other. Because the values were bigger at 580 nm, we represented these values.

### 2.3. Cell Enumeration

For the assessment of cell concentration, a Burker counting chamber was used. The depth of the counting chamber was 0.1 mm, and the counting area was 0.04 mm^2^. Cells were counted daily under the microscope (Nikon Eclipse Ni-E, Nikon Corporation, Tokyo, Japan). The cell concentration was calculated at a 250 × 1000 × dilution rate (cell number/mL).

### 2.4. Dry Mass Measurement

The dry weight (g/L) was monitored daily by filtering an aliquot of the culture (5–10 mL depending on the density) with pre-weighted glass microfiber filters (Whatman, Darmstadt, Germany, GF/C, 24 mm, pore size 1.2 μm). The filter was then dried at 80 °C for 5 h or to constant weight, prior to gross weight determination.

### 2.5. Determination of Colony Forming Units (CFU)

The cell suspensions of the bacterial cultures were serially diluted with sterile growth medium. Aliquots (0.1 mL) of the adequate dilutions were mixed with the melted (35 °C) soft agarose (2 mL) and poured onto the pre-warmed solid bottom agar in Petri dishes. The plates were incubated at 29 °C under continuous illumination at 30 μmol photons m^−2^ s^−1^ light intensity in the case of *S. elongatus* and in dark at 37 °C in the case of *E. coli*. The *E. coli* plates were evaluated the next day. After 3 weeks of incubation of *S. elongatus* plates, the number of colonies was counted. All the experiments were performed in triplicate, and results are expressed as an average of the triplicate samples with standard deviation.

### 2.6. Dye Exclusion Methods

If cells taking up trypan blue or propidium iodide (Merck/SigmaAldrich, Budapest, Hungary) are considered non-viable. We followed the manufacturer’s suggestions, with minor modifications. Shortly, 0.4% solution of trypan blue was prepared in BG11 medium buffered isotonic salt solution at pH 7.5. An amount of 0.1 mL of trypan blue stock solution was administered to 0.1 mL of cells, and it was examined immediately under a microscope at low magnification. An amount of 10 mg/mL propidium iodide was administered to *E. coli* cells. The detection happened at 540/490 nm excitation. The number of the total and that of the blue-stained or red fluorescent cells were characterized.

### 2.7. Fluorescent Microscopy

A Nikon Eclipse Ni-E (Nikon Corporation, Tokyo, Japan) was complemented with a CoolLED PE-4000 light source. Micrographs were taken by a Nikon D750 camera (Nikon Corporation, Japan).

### 2.8. Proteomics Analysis

#### 2.8.1. Sample Preparation for Affinity-Based Proteomics

Total proteins from *S. elongatus* were extracted, as described [22]. Total protein extracts (4 mg/IP) were immunopurified using anti-GFP antibody-coupled magnetic beads of 50 nm (MACS^®^ Technology, Miltenyi, Bergisch Gladbach, Germany), digested in column with trypsin and analyzed in a single run on the mass spectrometer [23]. The resulting tryptic peptide mixture was desalted prior to LC-MS/MS analysis on a C18 ZipTip (Omix C18 100 μL tips, Varian, Santa Clara, CA, USA), and the purified peptide mixture was analyzed by LC-MS/MS using a nanoflow RP-HPLC (LC program: linear gradient of 3–40% B in 100 min, solvent A: 0.1% formic acid in water, solvent B: 0.1% formic acid in acetonitrile), which was on-line coupled to a linear ion trap Orbitrap (Orbitrap-Fusion Lumos, Thermo Fisher Scientific, Waltham, MA, USA) mass spectrometer operating in positive ion mode. Data acquisition was carried out in a data-dependent fashion, and the 20 most abundant, multiply charged ions were selected from each MS survey for MS/MS analysis (MS spectra were acquired in the Orbitrap, and CID spectra were acquired in the linear ion trap).

#### 2.8.2. Data Interpretation

Raw data were converted into peak lists using the in-house Proteome Discoverer (v1.4) and searched against the Uniprot *Synechococcus elongatus PCC7942* (Syne7) database (downloaded 12 June 2019, 2653 proteins) using our in-cloud Protein Prospector search engine (v5.15.1) with the following parameters: enzyme: trypsin, with a maximum of 2 missed cleavages; mass accuracies: 5 ppm for precursor ions and 0.6 Da for fragment ions (both monoisotopic); fixed modification: carbamidomethylation of Cys residues; variable modifications: acetylation of protein N-termini; Met oxidation; cyclization of N-terminal Gln residues, allowing a maximum of 2 variable modifications per peptide. Acceptance criteria: minimum scores: 22 and 15; maximum E values: 0.01 and 0.05 for protein and peptide identifications, respectively. Spectral counting was used to estimate relative abundance of individual proteins in the non-antibody and GFP negative controls and in the anti-GFP immuno-purified samples.

## 3. Results

### 3.1. Characterization of the Growth of the Bacterial Population

**Figure 1:**
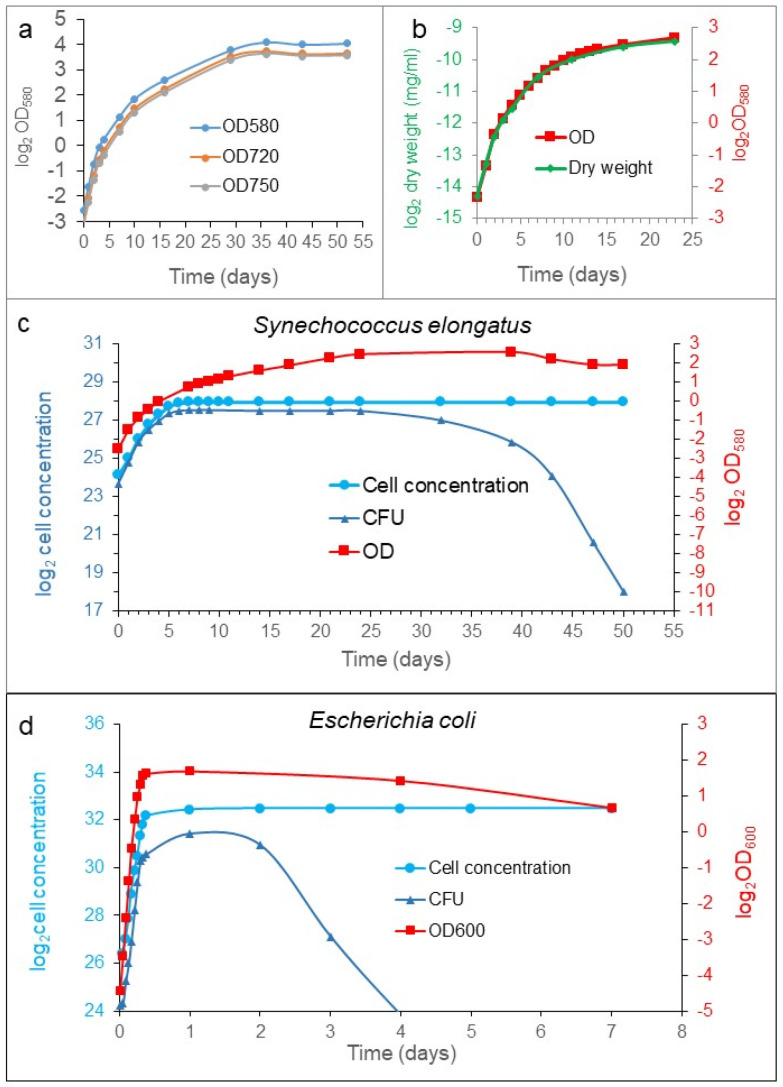
Characterization of population growth. *S. elongatus* cells were inoculated into BG11 medium, and the growth was monitored (**a**) at 580, 720, and 750 nm. Because there was not alteration in the shape of the curves, all the measurements happened at 580 nm. (**b**) Dry weight (left axis) and optical density (OD_580 nm_, right axis) are presented. (**c**) Cell enumeration and CFU (left axis), as well as optical density (right axis), can be seen in the case of *S. elongates*. The former experimental arrangement is shown in panel (**d**) in the case of *E. coli* without extra nutrients. The optical density and the dry weight change similarly. In the exponential phase, the cell concentration and the CFU steeply increase, reaching their plateaus; after a while, the CFU curve decreases sharply. Meanwhile, the cell concentration remains constant. A single point in the curves is the average of ten independent replicates, and the curves represent at least three independent cell cultivation experiments. All of the experiments were repeated three times. In all cases, the experiments were carried out in rich medium, and the *S. elongatus* populations were refed with nutrients in every 5th day until the 30th day. The standard errors of the individual points were characterized and are shown in Table S1 of the Supplementary Materials.

To monitor bacterial cell growth, different measurements can be used, such as optical density (OD), dry weight, bacterial enumeration (cell counting), and colony-forming ability (CFU). We analyzed the growth of *S. elongatus* and *Escherichia coli* populations in batch culture. We compared the growth curves determined with different methods. All the processes characterized the exponential phase similarly (Figure 1). However, they diverged from each other significantly from the transition between the exponential and stationary phases (Figure 1c). The stationary phase is defined as constant cell concentration, so the cell concentration of the population was halted upon reaching the plateau, as it was expected. Meanwhile, the OD and the dry weight kept on increasing in rich medium, which means that the cells were elongating and not dividing (see later). However, the CFU showed a robust difference from the previous ones during the examined period. In the early exponential phase, the CFU curve moved similarly as the cell concentration. However, in the transition phase between the exponential and stationary phases, the increment was decelerated, later stopped, showing a plateau, and then it was dropping down steeply to reach a very low value characteristic for the organism and the medium (Figure 1c); sometimes, it reached several ppm (parts pro mill) of the stationary cell concentration. The exhaustion of the nutrient must influence the CFU value in time, obviously. However, the decrease in CFU we observed occurred with a supplemented fresh stocks of nutrients.

Taken together, four ways of characterizations gave similar results for the exponential phase, but they became different from each other, starting from the transition phase. The cell concentration was more or less constant, OD values and dry weight increased parallelly, and the CFU had a maximum plateau at the transition phase. Then, it dropped down suddenly in the stationary phase, achieving a low, but constant, value for a while. The so-called death phase could not be detected with dye exclusion experiments during the investigation (three months) in well-fed cultures. The reduction in the colony-forming cells do not mean cell death, necessarily.

### 3.2. The Stationary Phase Is Neither Due to Nutrient Deprivation Nor Toxins

**Figure 2:**
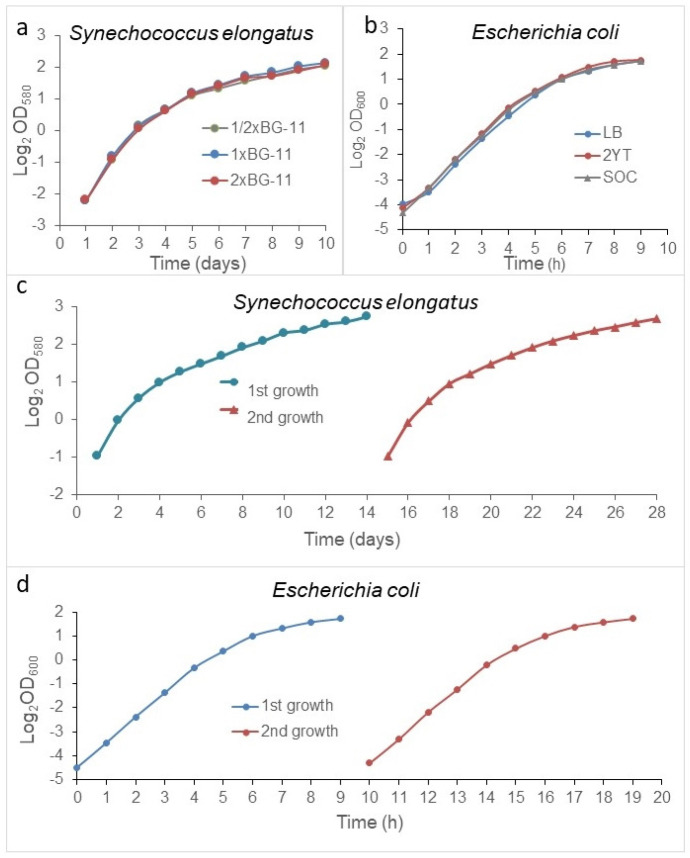
The stationary phase is not due to the exhaustion of medium or toxin. *S. elongatus* was grown (**a**) in 1/2×, 1×, and 2× BG11, and *E. coli* was cultivated (**b**) in LB, 2YT, and SOC media in batch cultures. *S. elongatus* was grown in a normal and spent medium (**c**), and *E. coli* population growths were tested in fresh and spent SOC media (**d**). The richness of media did not have any effect on the growth rate or the plateau. Cultivation of *E. coli* populations in spent media did not affect these parameters, either. The standard errors of the individual points were characterized and are shown in Table S1 of the Supplementary Materials.

According to long standing theories, the stationary phase is due to nutrient depletion and/or some toxin accumulation. We do not argue that it can happen, however, in lab conditions. Usually, nutrition deprivation rarely occurs. To prove this, *S. elongatus* cells were inoculated into 1/2×, 1×, or 2× BG11 media, setting the starting cell density to 0.2 OD_580_ (10 × 10^6^ cells/mL), and *E. coli* cells were inoculated into Luria-Bertani (LB) broth, 2YT, and SOC media. The extinction of the cultures was monitored daily at 580 nm and hourly at 600 nm for *E. coli*. In all media, there were no significant differences among the growth curves (Figure 2a,b). Furthermore, we tested whether toxic metabolites could accumulate during the growth phase. To achieve this, *S. elongatus* populations were grown in regular batch culture and in a special batch culture, wherein the medium was changed daily. The cell concentration of the cultures was monitored daily in a Burker chamber. No differences were detected in the growth rate and in the stationary plateau, either (Supplementary Figure S1).

We were interested in how the population would grow in spent medium. Therefore, the population was grown in a batch culture after reaching the stationary phase at ~4 OD_580_ for *S. elongatus* and ~3 OD for *E. coli.* The cells were removed by centrifugation, and the cell free, spent media were re-inoculated, adjusted with the stationary phase culture to ~0.5 OD_580_ in the case of *S. elongatus,* and, with regard to the original stationary phase culture related to 0.04 OD_600_ in the case of *E. coli* (again), the bacterial growth was followed. The population also reached ~4 OD_580_/3 OD_600_ in both cases after the second inoculation in the spent medium, too (Figure 2c,d). We repeated these experiments at least three times in both cases.

Importantly, the cultures reached the same plateau in spent medium, and in enriched medium, as well. It means that there was no toxin, and no deprivation of nutrient that could affect the growth rate during the exponential phase or the cell concentration of the stationary plateau. Further, the richness of media did not affect the stationary cell concentrations. Therefore, we concluded that the regulation of the transition from the exponential to the stationary phase was due to something other than exhaustion of media or toxins.

### 3.3. Characterization of the Stationary Phase; the Minimal Stationary Cell Concentration

**Figure 3:**
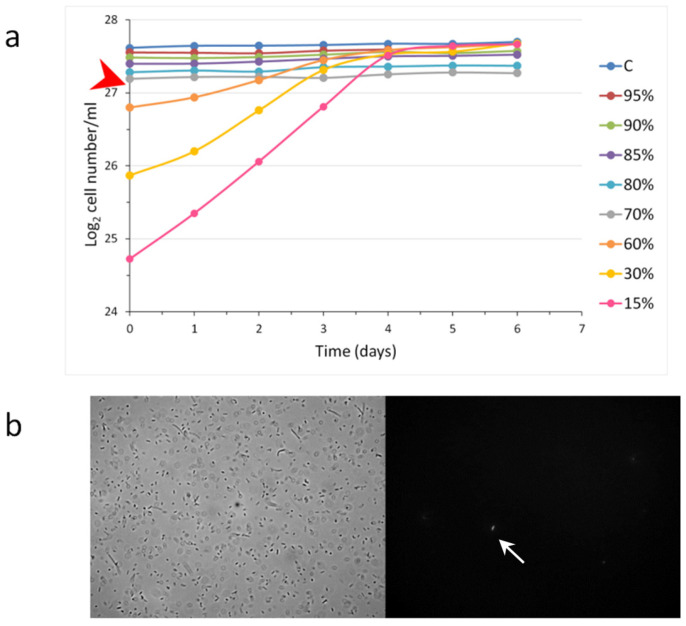
Characterization of the minimal stationary cell concentration (MSCC) (**a**). A stationery *S. elongatus* culture was diluted to the given percentage in fresh BG11 and used as an inoculate. In the case of *S. elongatus,* until 70% of the starting stationary cell concentration, the cell concentration of the populations did not change. However, 60% of the original cell concentration began proliferating and reached the starting stationary cell concentration. The MSCC is pointed by a red arrowhead. The standard errors of the individual points were characterized and are shown in the Table S1 of the Supplementary Materials. A dye exclusion experiment in *E. coli* cells with (10 mg/mL) propidium iodide shows that cell death could hardly be detected at the stationary phase (**b**). The micrographs were taken with 100× objectives, and the propidium iodide excitation happened at 540 nm. The propidium iodide uptake is pointed out by a white arrow.

We wanted to see how the growth of a population was influenced by the different starting cell concentrations used for inoculation. Therefore, different dilutions of stationary populations of *S. elongatus* were administered into fresh media, and the population growth was monitored (Figure 3). Interestingly we found that, when the inoculation happened with 90%, 80%, and 70% of the original stationary cell concentration, the cell concentrations remained almost still. At dilution of the stationary phase cells at 60% or below, all populations grew, reaching normal stationary cell concentrations (~220 × 10^6^/mL). This means that there was a limit to the dilutions of the non-growing populations that was considered a minimal stationary cell concentration (MSCC). This experiment could unravel the nature of the transition from the exponential to the stationary phase (Figure 3).

There was an additional result, namely, the dilutions closer to the MSCC originated flatter exponential curves, which means that the generation times are longer and longer. Importantly, the generation time should not be considered as a constant value. In the case of higher dilutions (e.g., 10×, 20×), the differences were not so significant.

According to the state-of-the-art, there are two possible models to explain the nature of the stationary phase: (1) the stationary phase would be due to a dynamic steady state of the continuous division and cells death or (2) contact inhibition among cells.

Dye exclusion experiments were performed to monitor whether continuous cell proliferation and death dynamically maintain a constant cell concentration of the stationary phase in *E. coli*. Trypan blue and propidium iodide were administered to the cultures. Theoretically, the living, intact cells exclude the dyes, and only dead cells absorb dyes. Neither significant cell proliferation, nor cell death, were detected during the period of the investigation corroborated by dye exclusion experiment. There was hardly any sign of cell destruction in the stationary population. Approximately 2 × 10^5^ cells were monitored, and ~5 ppm and ~27 ppm dead *E. coli* cells were detected with trypan blue and propidium iodide, respectively.

Contact inhibition could also be excluded because dilutions did not induce cell proliferation until the MSCC. This dilution experiment was executed with other prokaryotic and eukaryotic unicellular organisms, too, and the MSCC could be identified in the examined cases. For ease of comparison between different microorganisms, the ratio of MSCC to stationary cell concentration was investigated. This value was characteristic for the different organisms, including eukaryotic unicellular ones, as well (Table 1).

Taken together, in the case of the *S. elongatus,* we could find a minimal stationary cell concentration (MSCC), defined as a limit value of concentration as far as the diluted populations were still. However, below it, the population grew up. This value was ~70% of the native stationary cell concentration in the applied medium in the case of *S. elongatus*. Further, the stationary phase is not due to a dynamic equilibrium of a continuous cell division and death. MSCC could only be determined by serial dilutions of stationary-phase cells used as inoculates.

### 3.4. The Bacterial Cell Concentration Alone Is Not Enough to Set the Stationary Phase

**Figure 4:**
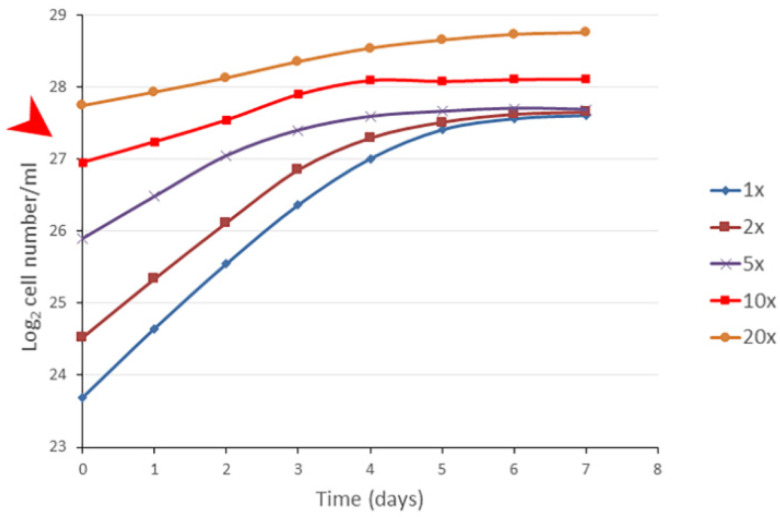
The cell concentration itself is necessary, but not sufficient, alone, to regulate the stationary cell concentration. An exponential phase *S. elongatus* culture (25 × 10^6^ cells/mL) was concentrated 1-, 2-, 5-, 10-, and 20-fold in fresh BG11 (25, 50, 75, 250, and 450 × 10^6^/mL), as it is stated in the figure. When assessing the MSCC (pointed out by the red arrowhead), the population slowly grew, and it roughly doubled the initial cell concentrations, while the populations below MSCC reached the native stationary cell concentration. Importantly, the generation time depended on the concentration of the inoculates. The standard errors of the individual points were characterized and are shown in Table S1 of the Supplementary Materials.

Exponential phase cells (~25 × 10^6^/mL) were concentrated by centrifugation and adjusted to the concentration of 25, 50, 75, 250, and 450 × 10^6^/mL, and they were suspended in fresh BG11 to check the population growth. If the inoculates were below the MSCC value, the population reached the original (native) plateau of stationary phase; where the cell concentration reached or exceeded the MSCC value, then the cell concentration approximately doubled, and proliferation stopped (Figure 4). The doubling mechanism was independent from the age of the exponential populations applied for the concentration. Interestingly, the growth rate of the concentrated populations was altered, decreasing with higher concentrations, meaning that the so-called generation time (doubling time of the given population) cannot be a constant value. It depended on the inoculating concentrations. Importantly, our statement is for populations, not for individual cell proliferation.

Taken together, if exponential phase cells were concentrated, they began proliferating no matter the concentration. However, the MSCC seemed a special point. Importantly, the cell divisions were not stopped by the cell concentration alone, meaning that the cell concentration by oneself is not enough to set the stationary phase.

### 3.5. The Stationary Phase Cells Are Different from the Dividing Cells

**Figure 5:**
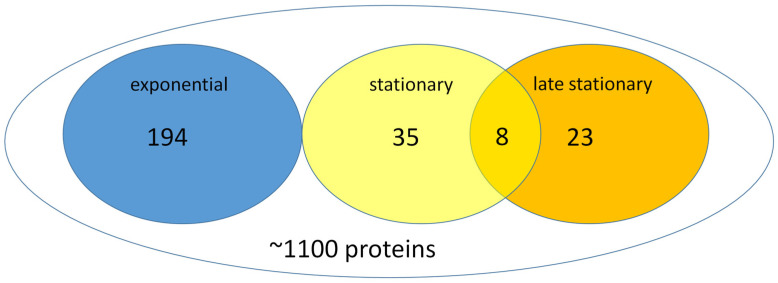
The results of the proteomic analysis. Affinity-based proteomic analysis was carried out with FtsZ-GFP as bait in all phases; the *S. elongatus* cells were grown in rich media. The results are presented in a Venn diagram; proteins characteristic only for the exponential phase are 194; for the stationary phase, there are 43, and, for the late phase, there are 31, and the early and late stationary phases shared eight proteins. The analysis is a result of three repetitions.

It was believed for a long time that, upon starvation, the eubacteria can enter a developmental program that results in metabolically different, more resistant cells [6]. Proteomic analyses were carried out on the *S. elongatus* cells of exponential, stationary, and late stationary phases to unravel the difference. The total protein content of the exponential-phase cells was five-fold higher than that of stationary-phase cells. Therefore, prior the analysis, it was normalized. We performed a FtsZ-GFP-based affinity proteomic analysis to monitor the alterations between the exponential and stationary phases. FtsZ interacted with approximately ~1100 proteins of a total (S1) of ~2660 proteins of *S. elongatus*, and, thus, we could exclude the very abundant photosynthetic proteins, which would overload the mass spectrometric detection. We could classify the detected proteins into four groups: (I) proteins whose expression did not change during the phases, considered constitutive proteins (Table S2 of Supplementary), (II) a group containing those proteins that were characteristic for the exponential phase, (III) a group comprised of the proteins typical for the stationary phase, and (IV) is characteristic by the late stationary phase (Figure 5). Table 2 shows the most important differences in the proteomics among the exponential and stationary and late stationary phases that we detected.

It has long been known that the protein composition of ribosomes is a sensitive sensor for the bacterial phases [24]. Well marked and characterized changes in the alternative use of some ribosome proteins are known, e.g., S6, S21, L12, L31, and L36 in *E. coli* [25,26,27,28]. In our experiments, all of the ribosomal proteins could be pulled down from the exponential lysate, so the ribosomal heterogeneity was able to be checked. S6, L7/12, L18, L20, and L29 proteins were missing or significantly under-represented in the stationary phase in *S. elongatus* (Table 2).

Without the claim of completeness, the proteomic data indicated that proteins involved in DNA replication and repair (e.g., helicases, gyrases, topoisomerases) were missing or underrepresented in the stationary phase (Table 2; Table S2 of Supplementary). Interestingly, well known cell division proteins, or proteins involved in cytokinesis, could be found with similar intensity in all phases, e.g., CDV3, MinD, Ftn2, etc. (Table 2.)

**Figure 6:**
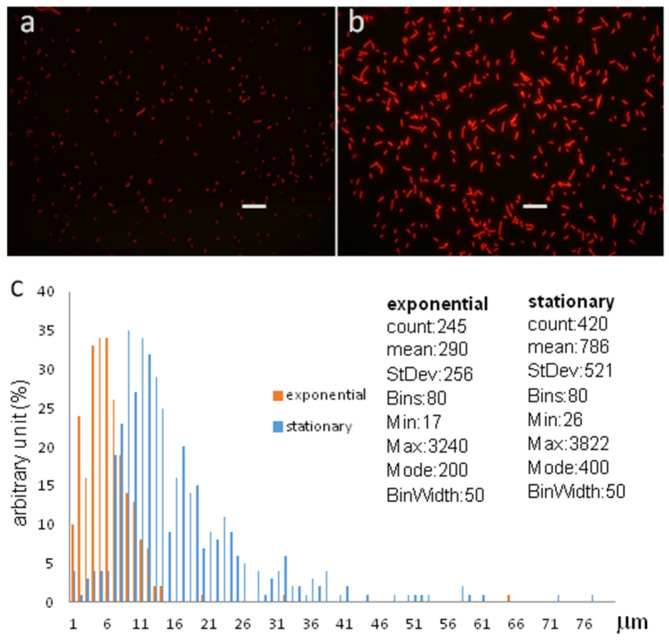
Phenotypic characterization of the stationary phase *S. elongatus* cells. The exponential-phase cells (**a**) had fainter auto-fluorescence upon 525 nm excitation than the stationary-phase cells (**b**), whose auto-florescence was much stronger. The pictures were taken with the same photo adjustments. Bars represent 10 μm. The size distribution of the two populations characterized by ImageJ (**c**) shows that the average exponential phase cells are 2.5-fold shorter than the differentiated, stationary phase cells. The vertical axis represents the number of bacteria, and the horizontal axis channels show the size of bacteria.

According to the data, we strongly believe that the stationary population consisted of differentiated cells, and a part of the population lost the capability of CFU. The proliferating populations could be distinguished from the stationary phase cells by phenotypic characters (Figure 6) and by proteomics. Proteomics of the late stationary phase cells (three months old) were altered very much from the exponential, and they were less altered than the early stationary phase cells (Table 2).

Taken together, there were characteristic proteomic differences among the studied groups that corroborated that genetic regulation controls the change in the phenotype to stationary state. According to our new model, the exponential phase cells [Exp] differentiate to stationary [Stat], then to late stationary, and those are called VBNC cells (cells that do not have CFU); this is the bacterial G0 in our nomenclature: [Exp]→[Stat]→G0.

### 3.6. Altered Phenotypes of Exponential Phase and Stationary-Phase Cells

Using fluorescence microscopy, the native, exponentially dividing *S. elongatus* cells could be distinguished from the differentiated non-proliferative cells by their size and auto-fluorescence at 525 nm excitation (Figure 6). Since the inoculating culture necessarily contained stationary G0 cells, the presence of stationary phase cells in the early population were not surprising; with the development of the exponential population, the concentration of the stationary phase cells decreased, and it was hardly detectable for a while.

## 4. Discussion

We examined the growth of the *S. elongatus* population in rich medium, focusing on the transition between the exponential and stationary phases. Our major novel findings are as follows: (1) the development of cell concentration of the stationary phase was not due to the exhaustion of nutrients or toxin(s). (2) The cell concentration is an important factor to stop cell proliferation. However, alone, it was not enough to set the stationary cell concentration. (3) We could define the so-called minimal stationary cell concentration (MSCC point), which refers to a cell concentration, as far as the diluted population of the native stationary phase remained constant. (4) The generation time was not a fixed value, rather, it depended on the inoculating concentrations. (5) The cell differentiation of a unicellular culture is an inevitable process, and it is genetically programmed; it can be characterized by [Exp]→[Stat]→G0 (VBNC). (6) In the stationary phase population, the phenotype of the cells was changed, and only the CFU started decreasing.

### 4.1. Characterization of the Growth of a Batch Culture

The growths of the populations were characterized in four ways: cell concentration (cell count/mL), dry weight (mg/mL), CFU (colonies/mL), and the optical density measurements. The curves separated from each other during the transition state (Figure 1). There was no sign of dead cells or debris in the stationary phase population. The so-called death phase could not be detected, even in late stationery cultures. However, a sharp decrement was found in the CFU at the stationary phase that might be confused frequently with the total cell concentration curve [29]. It may mean that the population loses its proliferating ability, most probably because of bacterial differentiation, but the cells are alive. Importantly, all of our experiments were executed in fresh, rich media.

### 4.2. Bacterial Differentiation

Our data emphasized that a bacterial population does not consist of continuously dividing and dying cells. Rather, it can be imagined as a complex community of bacterial cells behaving like a tissue. The proliferating cells [Exp] serve as stem cells. The role of this type of cells is to ensure a very rapid proliferation of the population to reach the maximum cell concentration characteristic for the stationary phase, determined genetically. The population of the stationary phase can be characterized as the body of the population comprising differentiated cells [Stat]. The stationary phase cells can be characterized as persistent cells, referring to a subpopulation of non-growing bacteria that are able to survive a high concentration of an antibiotic [30,31]. The [Stat] cells can still differentiate further to a G0, or VBNC form. The development of a prokaryotic culture can be described as [Exp]→[Stat]→G0/VBNC.

According to proteomics of the exponential and stationary populations of *S. elongatus,* there were several proteins, which were detected exclusively in one of the two populations that is we are able to distinguish between the pure dividing cells and stationary differentiated cells. In *E. coli,* there was a comparison between dividing and stationary-phase cells however, in this experiment, the stationary phase cells were starved, unfortunately [32]. It should be noted that, in the literature, usually starving cell populations are described upon examining the stationary phase [33]. A well acknowledged indicator of the stationary phase is the ribosome hibernating factor [34,35], which was present in both growth phases in *S. elongatus*, similarly to the Gram-positive *Staphylococcus aureus* [36]. This means that its expression could be regulated by starvation, rather than the native stationary differentiation.

### 4.3. Importance of the MSCC

If stationary populations were diluted for inoculation, there was a range where the population did not commence dividing, and the cell concentration remained constant. The percentage of MSCC over stationary cell concentration is a characteristic value for the different unicellular strains, which alters in a rather wide range (Table 1). It seems this feature is evolutionary conserved among the unicellular organisms and can have evolutionary advantages. Here, we do not discuss the importance of the MSCC for the microbiome and biotech applications because it goes beyond this manuscript. However, it is worth considering this novelty for future research.

### 4.4. The Generation Time Is Dependent on Cell Concentration

Interestingly, the growth rate depended on the inoculating cell concentration. Although the cells used as the inoculate originated from a stationary population, containing partly non-proliferative cells, it would be plausible to explain the slower doubling time by the high rate of the differentiated G0-like cells. However, a similar phenomenon could be seen in the case of the concentrated exponential phase cells, as well (Figure 4). We concluded that the less the inoculating cell concentration was, the higher the growth rate was. This observation can explain the anomaly of growth rates measured in turbidostats [37].

The main question is how the stationary phase is formed and the cell concentration is held still elusive, but it must be genetically coded.

### 4.5. The Nature of the Stationary Phase

Although the state-of-the art still prefers a picture of continuously growing bacterial population with perpetual proliferation and death [13,29], our data clearly demonstrated that it does not happen. Rather, the total cell concentration can act as a brake for cell proliferation [33], and this braking effect could suppress individual cell divisions. Some brakes should be switched, most probably at the end of the exponential phase and when the population just finishes divisions, reaching the native stationary cell concentration. Importantly, the brake and the differentiation have a genetic origin, but the real trigger is enigmatic.

## 5. Conclusions

Here, we provide a novel interpretation of the regulation of the growth of bacterial populations. According to the new observations, the development of the stationary phase and the stationary cell concentration is genetically coded; well fed populations have the ability to regulate the divisions of the single cells. The stationary-phase cells are differentiated cells, most probably persisters, and a part of the population should be considered VBNC cells, which lose colony-forming capability. Therefore, a bacterial population should be considered as a complex community of cells similarly to a more developed organism with proliferative (stem cells) and non-proliferating (somatic) cells. A tissue-like behavior does not necessarily require direct cell-to-cell contact, and communication is much more important among cells than a physical connection.

## Figures and Tables

**Table 1:** Minimal stationary cell concentration (MSCC) in different organisms given in percentage of the dilutions of the stationary cell concentration, where the populations were still, and they did not grow.

*Synechococcus elongatus*	70%
*Escherichia coli*	30%
*Schizosaccharomyces pombe*	55%
*Chlorella vulgaris*	95%
*Anabaena* sp. *	95%
*Auxenochlorella protothecoides*	70%

* *Anabaena* sp. growth was characterized by its chlorophyll content.

**Table 2:** The results of the affinity-based proteomics of *S. elongatus* cells using FtsZ-GFP as bait. The list of all the pulled-down proteins can be found in Table S2 of the Supplementary section.

Known Proteins Are Involved in Cell Division and Cytokinesis							
Uniprot Code	Name	Number of Unique Peptides and Frequency, Respectively					
		Exponential		Stationary		Late Stationary	
* O85785	FtsZ	47	230	36	143	37	190
Q31RI7	MreB	45	88	34	51	38	71
Q31PU3	MinD	22	29	14	18	17	36
Q93AK0	Ftn2	27	38	15	16	22	43
Q31LN3	CDV3	5	5	7	8	4	4
Q31RR7	MinD2	8	9	7	8	6	17
**Proteins characteristic only for the exponential phase cells**							
Q55107	Bicarbonate transport	39		61			
Q8GIT7	DNA gyrase subu B	39		49			
Q31RN3	DNA gyrase subu A	21		22			
Q31M73	His kinase	26		26			
Q31P89	DnaA	20		25			
Q31RU3	DNA polI	14		14			
Q31K18	Penicillin-binding prot 1A	13		14			
Q31MB0	RecN DNA repair	10		10			
Q31KL2	FtsQ	8		8			
Q31PH9	rib. pseudouridine synth	8		9			
Q31SC5	30S rib prot S6	3		3			
Q31L22	50S rib. prot L18	3		4			
**Proteins characteristic only for the late stationary phase**							
Q31NF6	Inorganic pyrophosphatase	7		16			
Q31RU6	Ser-glyoxylate transaminase	5		6			
Q31PP8	ATPase	5		5			
Q55024	DNA protect. during starvation	4		4			
**Protein characteristic only for the stationary phase**							
Q31KE5	Phosphate binding protein	18		38			
P39665	SphX	15		20			
Q31N73	Cystein synthase	13		17			
Q31LV5	D-Ala-D-Ala carboxypeptidase	10		10			
Q31N35	Unchar. prot	10		10			

* FtsZ is supposed to be the bait of the pull-down experiment.

## Data Availability

Not applicable.

## Supplementary Materials

The following supporting information can be downloaded at: https://www.mdpi.com/article/10.3390/cells12101430/s1, Figure S1: The stationary phase is not due to exhaustion of medium; Table S1: The standard errors of the individual measurements; Table S2: Proteomic data of FtsZ-GFP pull-down affinity-based proteome.
