# Supplementary figures and images for: Reconsidering Dogmas about the Growth of Bacterial Populations
Source: Cells. 2023 May 19;12(10):1430. doi: 10.3390/cells12101430 (PMC10217356; doi:10.3390/cells12101430)

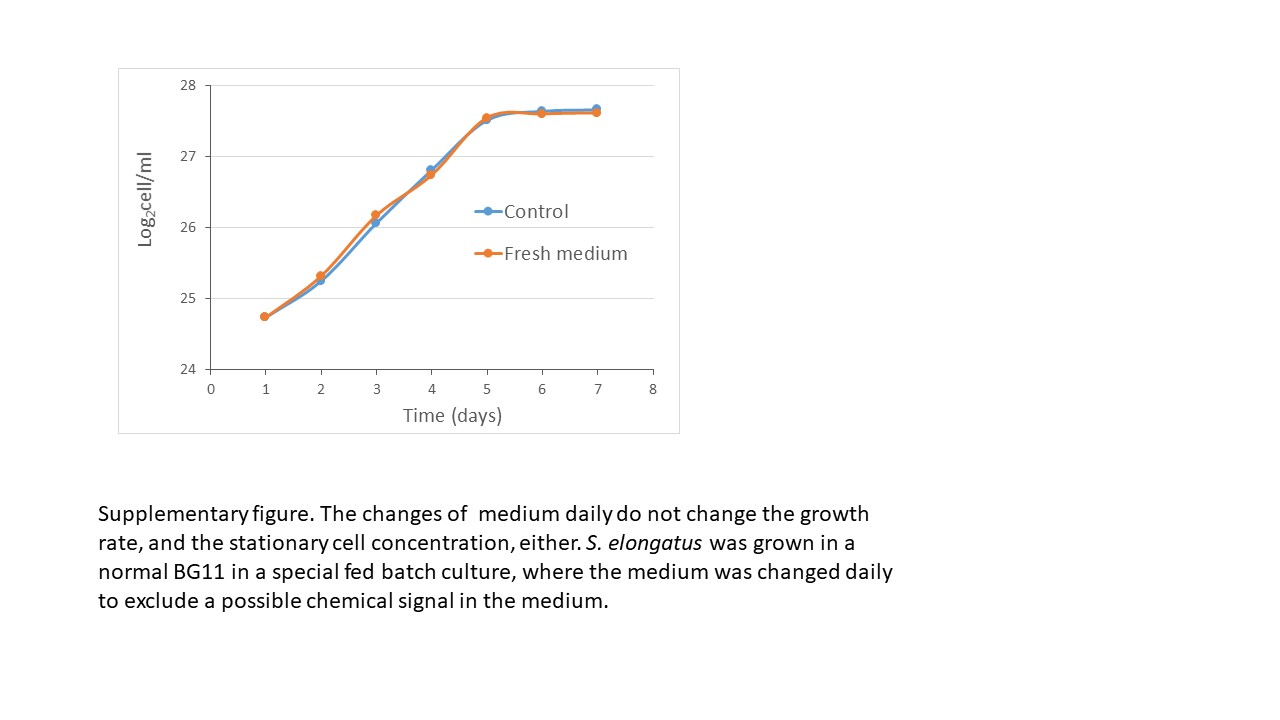

Supplement: Supplementary file 1 [file cells-12-01430-s001.zip › Suppl Fig upload.jpg]
